# Differences in stromal component of chordoma are associated with contrast enhancement in MRI and differential gene expression in RNA sequencing

**DOI:** 10.1038/s41598-022-20787-3

**Published:** 2022-10-03

**Authors:** Mina Park, Inho Park, Chang-Ki Hong, Se Hoon Kim, Yoon Jin Cha

**Affiliations:** 1 Department of Radiology, Gangnam Severance Hospital, Yonsei University College of Medicine, Seoul, South Korea; 2 Center for Precision Medicine, Gangnam Severance Hospital, Yonsei University College of Medicine, Seoul, South Korea; 3 Department of Pathology, Gangnam Severance Hospital, Yonsei University College of Medicine, Seoul, South Korea; 4 Department of Neurosurgery, Gangnam Severance Hospital, Yonsei University College of Medicine, Seoul, South Korea; 5 Department of Neurosurgery, Asan Medical Center, University of Ulsan College of Medicine, Seoul, South Korea; 6 Department of Pathology, Severance Hospital, Yonsei University College of Medicine, Seoul, South Korea

**Keywords:** CNS cancer, Genetics research, Translational research, Neuroscience, Biomarkers, Oncology

## Abstract

Chordoma is a malignant bone neoplasm demonstrating notochordal differentiation and it frequently involves axial skeleton. Most of chordomas are conventional type with varying amount of myxoid stroma. Previously known prognostic factors for conventional chordoma are not specific for chordoma: old age, metastasis, tumor extent, and respectability. Here, we aimed to investigate the histologic, radiologic, and transcriptomic differences in conventional chordoma based on the stromal component. A total of 45 patients diagnosed with conventional chordoma were selected between May 2011 and March 2020 from a single institution. Electronic medical records, pathology slides, and pretreatment magnetic resonance imaging (MRI) scans were reviewed. Of the 45 patients, ten cases (4 stroma-rich and 6 stroma-poor tumor) were selected for RNA sequencing, and available cases in the remainder were used for measuring target gene mRNA expression with qPCR for validation. Differential gene expression and gene set analysis were performed. Based on histologic evaluation, there were 25 (55.6%) stroma-rich and 20 (44.4%) stroma-poor cases. No clinical differences were found between the two groups. Radiologically, stroma-rich chordomas showed significant signal enhancement on MRI (72.4% vs 27.6%, *p* = 0.002). Upregulated genes in stroma-rich chordomas were cartilage-, collagen/extracellular matrix-, and tumor metastasis/progression-associated genes. Contrarily, tumor suppressor genes were downregulated in stroma-rich chordomas. On survival analysis, Kaplan–Meier plot was separated that showed inferior outcome of stroma-rich group, although statistically insignificant. In conclusion, the abundant stromal component of conventional chordoma enhanced well on MRI and possibly contributed to the biological aggressiveness that supported by transcriptomic characteristics. Further extensive investigation regarding radiologic-pathologic-transcriptomic correlation in conventional chordoma in a larger cohort could verify additional clinical significance.

## Introduction

Chordoma is an aggressive malignancy that categorized into bone tumor as it usually occurs in the axial skeleton. Phenotypically, chordoma recapitulates notochordal cells and has notochordal differentiation. Chordomas could be classified into three subtypes, based mainly on the histology and genetic characteristics: Conventional^1^, dedifferentiated^2,3^, and poorly differentiated chordomas^4^. Conventional chordoma is the most common type of chordoma, composed of large epithelioid tumor cells and occasional physaliphorous cells arranged in cords and nest, that embedded within a varying amount of extracellular myxoid and/or myxochondroid matrix. Conventional chordoma (hereafter chordoma) is genetically characterized by brachyury expression, a specific marker for notochordal differentiation^1,5^. Dedifferentiated chordoma is the rarest form of chordoma that has both conventional chordoma and high-grade sarcoma component^2,3^. Poorly differentiated chordoma is a rare and distinct subtype with loss of SMARCB1 expression and usually affects children^6^. Dedifferentiated and poorly differentiated forms are very rare, and have worse prognosis than conventional chordoma^7–9^.

Current treatment strategies for chordoma include surgical resection and adjuvant radiation, proton, and photon therapies; however, curative resection is often challenging due to the anatomical location of tumors, particularly for the skull base chordomas as they are closely packed with adjacent cranial nerves, brainstem, and skull base vasculature^10,11^. So far, various prognostic factors were proposed for skull base chordomas, including the extent of surgical resection, tumor size, preoperative Karnofsky Performance status, and type of adjuvant therapy^12–16^.

Skull base chordomas often demonstrate variable magnetic resonance imaging (MRI) features as chordomas may have a highly heterogeneous appearance demonstrating moderate to high intensity on T2 sequences and minimal to moderate contrast enhancement^17,18^. Several studies have revealed that the contrast enhancement of chordoma appeared to be a risk factor of tumor progression and/or recurrence^18,19^. The presence of enhancement in chordoma suggests that it might be associated with distinctive histopathologic and transcriptomic characteristics. A recent study calculated the tumor-stroma ratio (TSR) in spinal chordoma and found that predominant stromal component was related to inferior clinical outcome^20^. Considering that contrast enhancement and T2 signal intensity are the surrogates for the different tumor component, we speculated that stromal component of chordoma may be differently demonstrated, as well as has different transcriptomic characteristics. Herein, we aimed to investigate whether stromal amount in chordoma affects the radiologic features and transcriptomic differences to further clarify the clinical implication of conventional chordoma.

## Results

### Patient characteristics

The basal characteristics of patients (n = 45) are shown in Supplementary Table 1. The patients were diagnosed with chordoma at a mean age of 47 years (range 17–76 years) with a male to female ratio of 20:25. Of them, 31 had primary chordomas and 14 had recurrent tumors. The mean tumor size was 32.3 ± 19.0 mm. Among the patients, 28 (63.6%) achieved total tumor resections, while 16 (36.4%) had subtotal resections. Forty-two (93.3%) of the 45 patients had adjuvant radiation therapy, and radiologic follow-up was available for 43 patients, with a median follow-up time of 621 days (interquartile range 270.5–1768.3). Of the 43 patients, 15 (34.8%) exhibited disease progression.

### Comparisons of clinicoradiologic features according to stromal proportion

There were 25 (55.6%) stroma-rich and 20 (44.4%) stroma-poor chordomas. There were no significant differences between the two groups in terms of age, sex, tumor size, and presence of tumor progression (Supplementary Table 1). On the histologic evaluation, stroma-poor chordomas frequently had benign notochordal cell tumor (BNCT)-like component (65.0% vs 4.0%, *p* < 0.001) as well as high cellularity (90% vs 40%, *p* < 0.001) compared to the stroma-rich chordomas (Table 1).

**Table 1:** Comparison between histomorphologic and radiologic features according to the stromal component of chordomas.

Variables	Stroma-poor (n = 20)	Stroma-rich (n = 25)	*p*-value
**Histology (n, %)**			
Nuclear pleomorphism	4 (20.0%)	7 (28.0%)	0.729*
Necrosis	2 (10.0%)	1 (4.0%)	0.577*
BNCT-like component	13 (65.0%)	1 (4.0%)	< 0.001
Cellularity (%)	90 (IQR 72.5–90.0)	40 (IQR 20–60)	< 0.001^§^
**Imaging (n, %)**			
T2 signal intensity			1.000*
High	16 (80.0%)	21 (84.0%)	
Intermediate	4 (20.0%)	4 (16.0%)	
T2 signal homogeneity			0.182
Homogeneous	12 (60.0%)	10 (40.0%)	
Heterogeneous	8 (40.0%)	15 (60.0%)	
Enhancement, presence	8 (40.0%)	21 (84.0%)	0.002

*BNCT* benign notochordal cell tumor, *IQR* Interquartile range.

*Fisher’s exact test.

^§^Mann–Whitney test.

**Figure 1:**
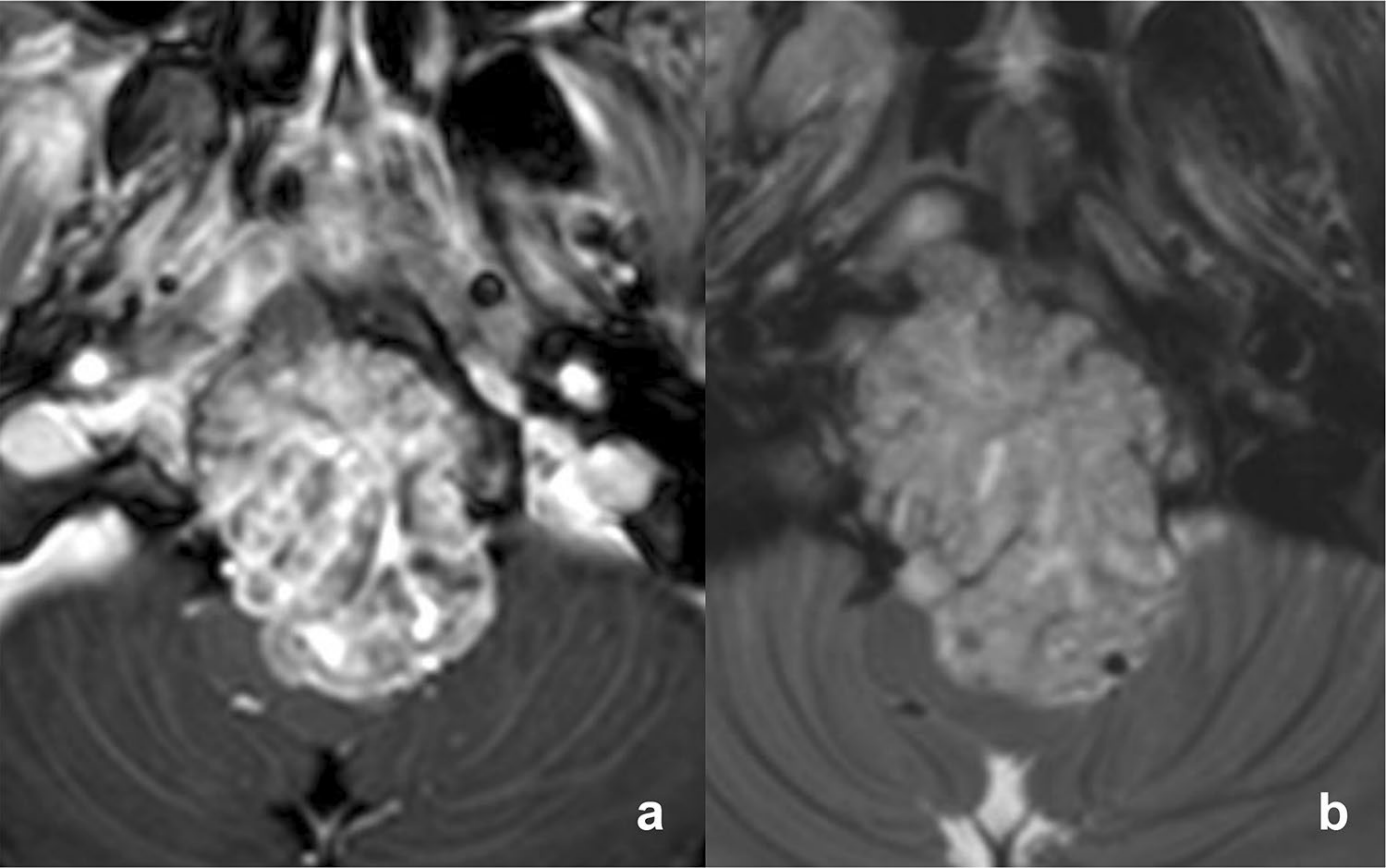
Magnetic resonance imaging of stroma-rich chordoma. (**a**) Contrast-enhanced T1-weighted and (**b**) T2-weighted imaging show an avidly enhancing expansile mass extending from the clivus with T2 intermediate signal intensity.

**Figure 2:**
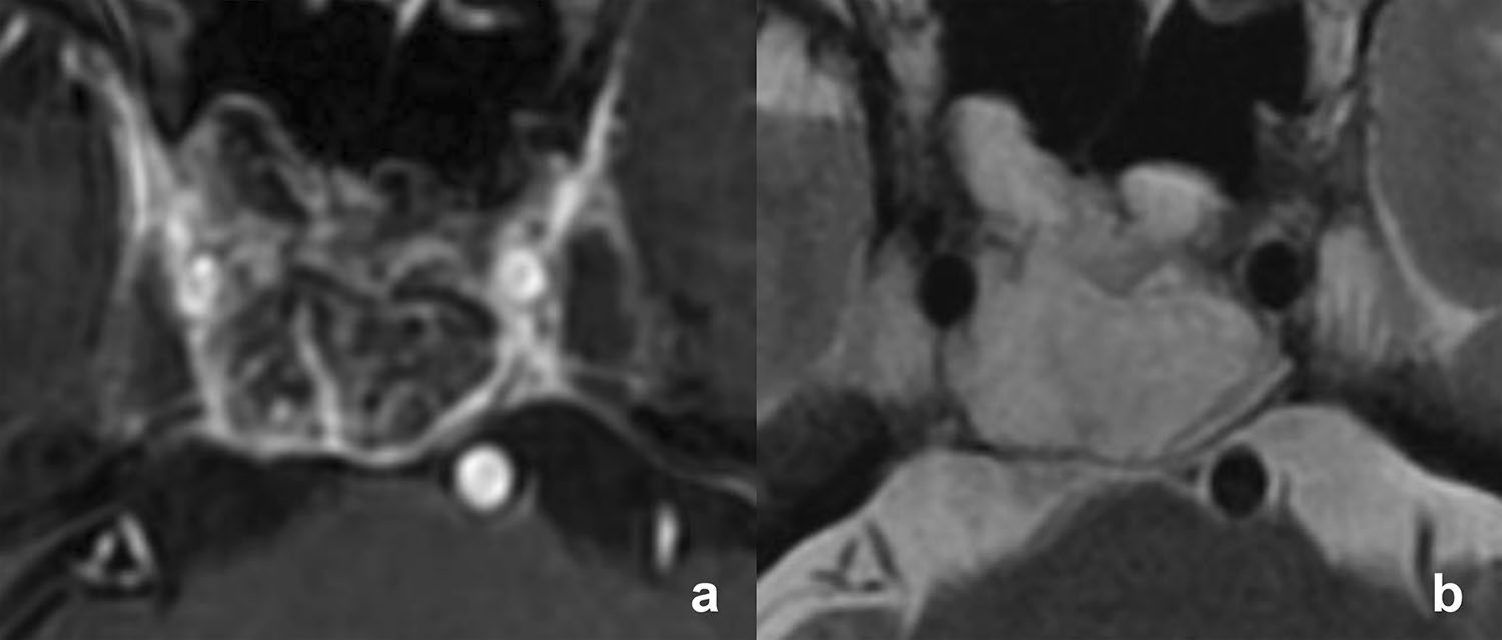
Magnetic resonance imaging of stroma-poor chordoma. (**a**) Contrast-enhanced T1-weighted and (**b**) T2-weighted imaging show a poorly enhancing mass originating from the clivus with T2 high signal intensity.

Among the radiologic findings, contrast enhancement on MRI was observed more frequently in the stroma-rich group (Fig. 1) than in the stroma-poor group (84.0% vs 40.0%, *p* = 0.002; Fig. 2, Table 1). T2 signal intensity and its homogeneity in the lesions did not exhibit significant difference. Patients with stroma-rich chordoma showed shorter progression-free survival (PFS) but did not reach to the statistical significance (1555 days vs. 2106 days, *p* = 0.4, Supplementary Fig. 1).

### Differentially expressed genes (DEGs) according to stromal proportion

**Figure 3:**
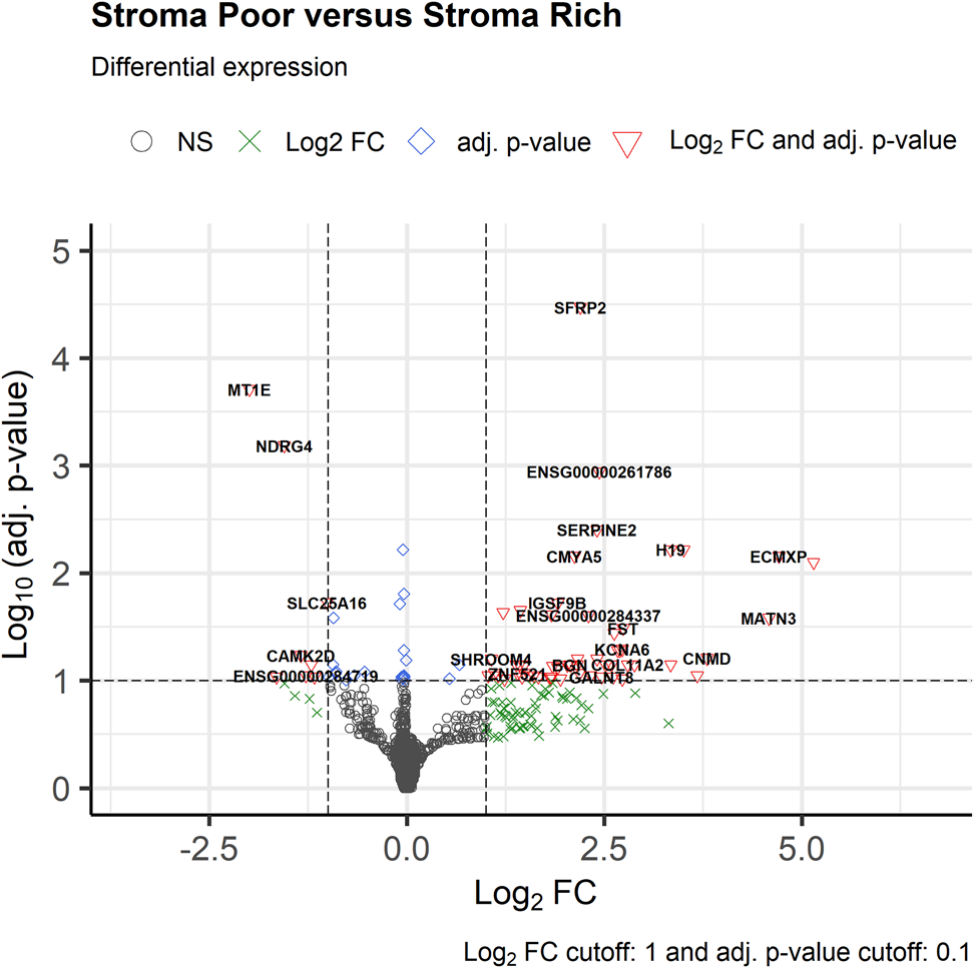
Volcano plot of the differentially expressed genes between stroma-rich and stroma-poor chordomas.

**Figure 4:**
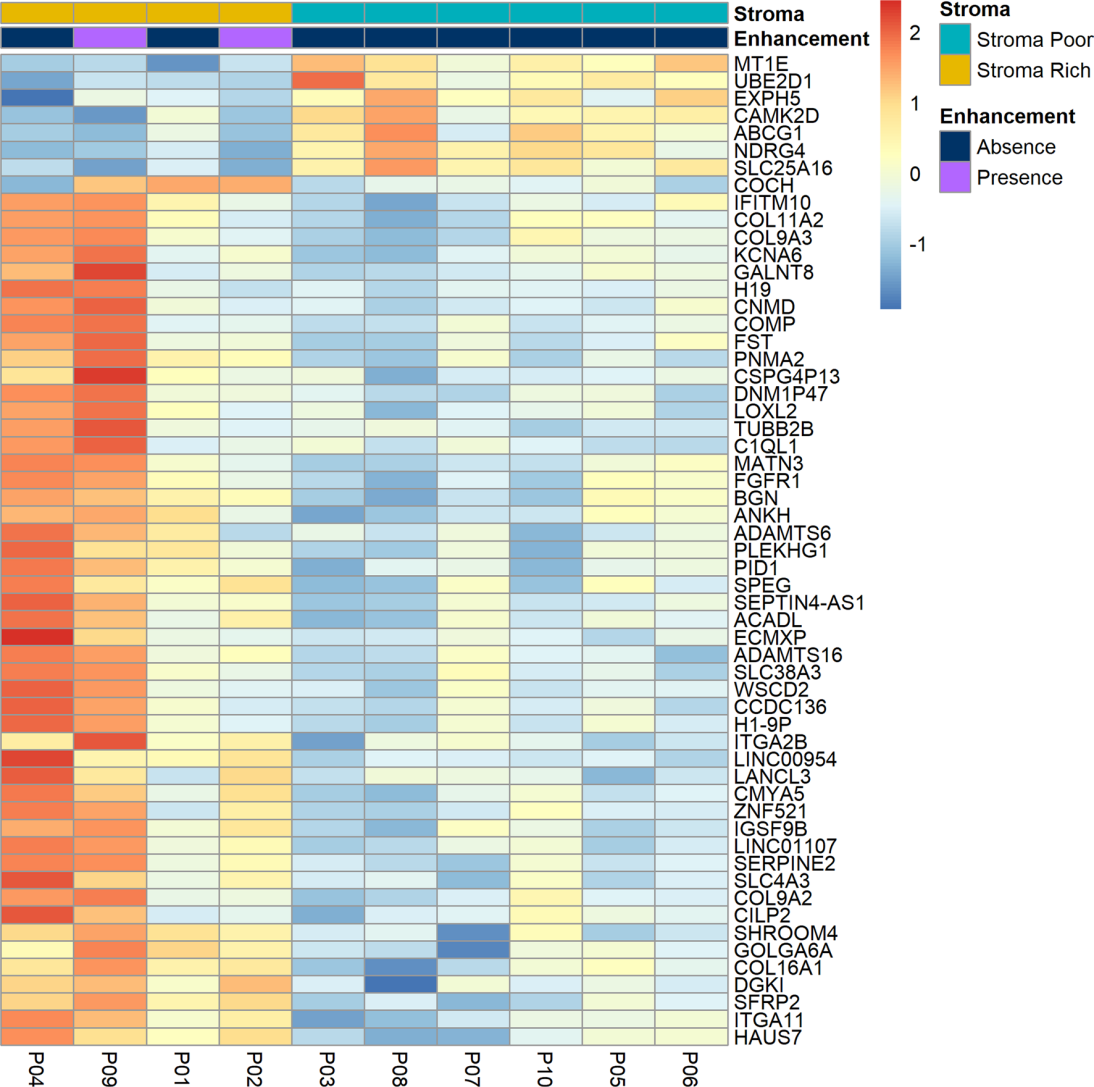
Heatmap view of the 10 patients with chordoma. Y-axis represents the differentially expressed genes; X-axis represents the patients with chordoma. The expression levels from low to high are represented as a color gradient from blue to red, respectively. There are two color bars of the heatmap to represent stroma status (stroma-rich and stroma-poor) and presence of enhancement on magnetic resonance imaging.

We compared the gene expression in stroma-rich (n = 4) and stroma-poor chordomas (n = 6) and found that 50 genes were significantly upregulated (log_2_ FC > 1 and adjusted *p* < 0.1) and seven genes were significantly downregulated (log_2_FC < 1 and adjusted *p* < 0.1) in stroma-rich chordomas (Table 2, Figs. 3, 4). Upregulated genes included cartilage-associated genes (*COMP*, *MATN3*, and *COL11A2*), collagen- and extracellular matrix-associated genes (*COL9A2*, *SFRP2*, and *SERPINE2*), and tumor metastasis- and progression-associated genes (*SLC38A3*, *FST*, *ITGA11*, and *DGKI*). Downregulated genes included previously known tumor suppressors in other organs, such as *NDRG4*, *MT1E*. On principal component analysis (PCA) plot using the 500 most variable genes did not reveal any significant bias from the dataset (Supplementary Fig. 2).

**Table 2:** Differentially expressed genes according to the stromal component of chordomas.

Gene	Description	Fold change	Adjusted *p*-value
**Up-regulated genes in stroma-rich chordomas (top 20 genes from total of 50 genes, adjusted *p*-value < 0.1, Fold change > 2.0)**			
SFRP2	Secreted frizzled related protein 2	4.59	< 0.001
SERPINE2	Serpin family E member 2	5.30	0.004
H19	H19 imprinted maternally expressed transcript	10.12	0.006
CMYA5	Cardiomyopathy associated 5	4.35	0.007
ECMXP	Extracellular matrix protein X-linked, pseudogene	26.13	0.007
COMP	Cartilage oligomeric matrix protein	35.45	0.008
IGSF9B	Immunoglobulin superfamily member 9B	3.76	0.019
PNMA2	PNMA family member 2	2.70	0.022
LINC00954	Long intergenic non-protein coding RNA 954	2.33	0.023
ITGA11	Integrin subunit alpha 11	3.56	0.024
MATN3	Matrilin 3	23.95	0.026
FST	Follistatin	6.68	0.033
ADAMTS16	ADAM metallopeptidase with thrombospondin type 1 motif 16	6.17	0.036
KCNA6	Potassium voltage-gated channel subfamily A member 6	6.63	0.051
COCH	Cochlin	6.48	0.052
CNMD	Chondromodulin	13.96	0.062
WSCD2	WSC domain containing 2	5.30	0.063
TUBB2B	Tubulin beta 2B class IIb	4.46	0.063
SHROOM4	Shroom family member 4	2.10	0.063
**Down-regulated genes in stroma-rich chordomas (adjusted *p*-value < 0.1, fold change < 0.5)**			
MT1E	Metallothionein 1E	0.25	< 0.001
NDRG4	NDRG family member 4	0.34	0.001
SLC25A16	Solute carrier family 25 member 16	0.50	0.019
CAMK2D	Calcium/calmodulin dependent protein kinase II delta	0.39	0.058
UBE2D1	Ubiquitin conjugating enzyme E2 D1	0.43	0.071
EXPH5	Exophilin 5	0.32	0.093
ABCG1	ATP binding cassette subfamily G member 1	0.44	0.093

Since all the samples are located within the 95% confidence ellipse of their respective group, we did not exclude any sample for DEG analysis.

**Figure 5:**
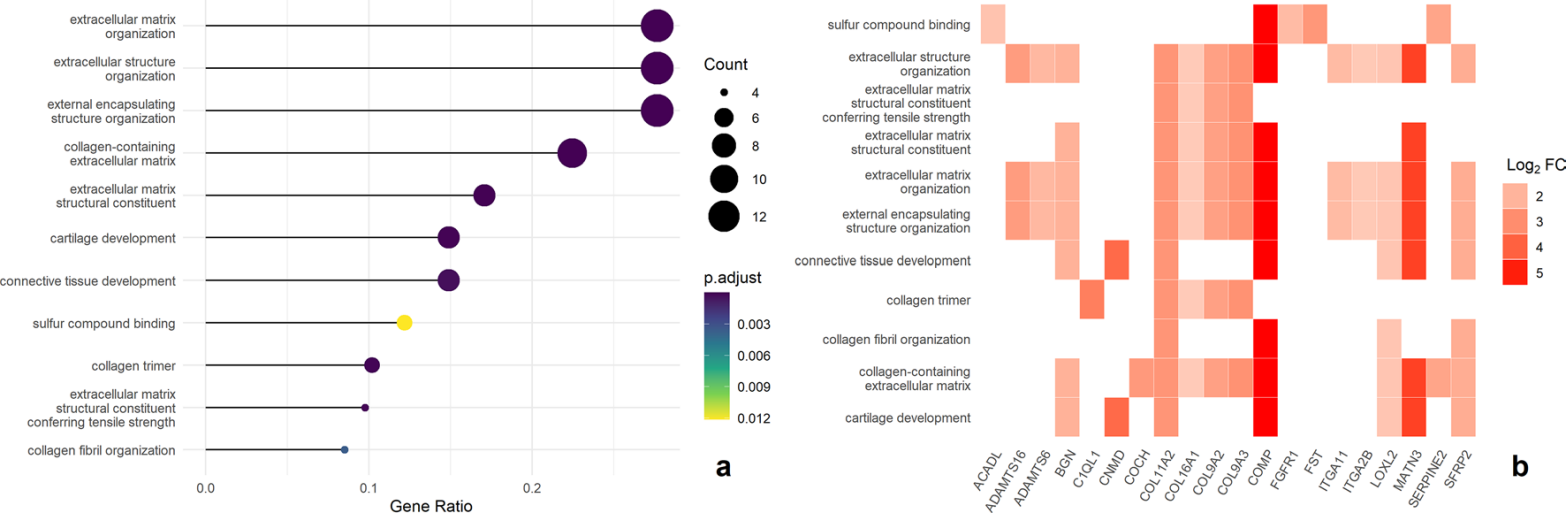
Enriched Gene Ontology terms (**a**) bubble plot for the enriched GO terms (**b**) heatplot for the enriched GO terms.

Gene set analysis of differentially expressed genes on gene ontology terms revealed that most of the significant gene expression in stroma-rich chordoma was associated with extracellular matrix organization, cartilage development, and collagen fibril organization (Fig. 5, Supplementary Table 2).

### Quantification of target gene mRNA expression

With the selected eight genes (four upregulated and four downregulated genes in RNA sequencing), mRNA quantification was performed in additional 30 samples. Samples with C_T_ value of *GAPDH* < 35 were included for analysis. Although it did not reach statistical significance, there was a tendency of difference in mRNA expression between stroma-poor and stroma-rich chordomas (Supplementary Fig. 3).

## Discussion

We investigated that the proportion of stromal component in chordoma affects radiologic features as well as gene expression profile. Stroma-rich chordomas showed frequent enhancement on MRI and had different transcriptomic profile from that of stroma-poor chordomas.

In this study, we only included conventional chordomas, as other two subtypes—dedifferentiated and poorly differentiated chordomas—are extremely rare and were thought to be difficult to derive statistically significant results. So far, genetic characteristics of chordoma has been investigated in respect of the intrinsic tumor characteristics, such as tandem duplication or copy-number gain of *TBXT*, encoding brachyury^1,5,21–23^. Myxochondroid stroma is one the distinct pathologic feature of chordoma. Due to the chondroid feature of stroma, one of important differential diagnosis of chordoma had been chondrosarcoma before brachyury was discovered^8,24^. According to the stromal proportion, chordoma could demonstrate variable spectrum of pathologic and radiologic features. Hence we speculated that stroma-rich and stroma-poor chordomas would show different clinical, radiologic and transcriptomic features.

Clinically, Kaplan–Meier curve of PFS was separated according to the stromal proportion, but statistical significance was not found. However, stroma-rich chordomas had lower cellularity and lesser BNCT-like component in histological aspect, and showed frequent contrast enhancement on MRI. This is an interesting finding because enhancement on MRI usually positively correlated with the cellularity of tumor, particularly tumor vasculature. Several studies reported that the presence of enhancement may be associated with poor prognosis in the skull base chordoma, none explained the histopathological background regarding the association between prognosis and contrast enhancement^19,25^. Previous study with skull base chordoma developed the MR grading system based on the tumor enhancement, and chordomas with abundant blood supply were more highly enhanced and showed worse PFS^18^. In this study, we observed that in stroma-poor chordoma, higher cellularity was commonly derived from the abundant BNCT-like component. BNCT-like component recapitulates the notochordal cells, which takes more differentiated form among the tumor cells that are densely packed with sparse vascularity. However, in this study, we could not quantitate the vasculature in chordoma. Considering that most of chordoma specimen is arrived at pathologic laboratory in fragmented form, accurate measurement and comparison of microvessel density of chordoma sample could be hardly performed, but still worth to investigate.

As expected, stroma-rich chordomas showed elevated expression of cartilage-associated genes, including *COMP*, *MATN3*, and *COL11A2*^26,27^. Noncartilaginous extracellular matrix-associated genes, including *COL9A2*, *SERPINE2*, and *ITGA11*, were also upregulated^28,29^. Interestingly, some genes that were upregulated in stroma-rich chordoma were associated with tumorigenesis, tumor progression, and poor clinical prognosis in other solid tumors. For instance, *SLC38A3* was reported to be upregulated in metastatic non-small cell lung cancer and related to poor prognosis^30^; *FST* is known to be associated with tumorigenesis, progression, metastasis, and angiogenesis of solid tumors^31^, and *SERPINE2* promoted local invasion of pancreatic tumor cells in vivo^28^. Most of the genes related to tumor progression, metastasis, and poor prognosis promote tumor cell invasiveness and migration, which are the signatures of epithelial-mesenchymal transition (EMT) of tumor. Moreover, as stroma-rich chordoma is enriched with the matrix component, mesenchymal signature might have been more prominent.

Recent study with spinal chordoma reported that low TSR of chordoma was associated with poor prognosis^20^. As low TSR means “stroma-rich”, their result is partly correlated with ours that stroma-rich chordoma tends to be more aggressive, and has upregulated genes which are associated with not only tumor metastasis and prognosis but also collagen and matrix. This implies that stroma-rich chordoma could have more predominant EMT feature, which is also associated with angiogenesis^32^.

Most of the upregulated genes in stroma-poor chordomas were tumor suppressor genes. However, *MT1E*, also a tumor suppressor, was recently revealed to be elevated in malignant astrocytoma^33^.

On further mRNA expression analysis, there was a tendency of differential quantitative mRNA expression that followed the results of DEG of RNA sequencing, although statistically insignificance. This might be explained by the intrinsic characteristics of chordoma and the specimen quality. Generally, chordoma is low cellular tumor and the specimen obtained from the skull base surgery is usually very small amount and mixed up with bony fragments, hemorrhage and fibrotic surrounding tissue. These mixed components might lower the tumor cellularity and influent on the RNA quality.

One of important limitation of this study is heterogeneity of cohort. Although all patients received standard treatment, patients with skull base chordoma could not achieve the curative resection in most case due to the complex anatomical structures, and various adjuvant treatments, which also might have effect on PFS. However, to focus on the tumor histology with small number of patients, further subgroup analysis was unavailable. Furthermore, target gene mRNA expression data was not sufficient to validate DEGs, even though we critically qualified the RNA quality prior to sequencing and analyzed all available samples. However, as mentioned above, tiny tumor specimen, low cellularity, and bony detritus may have affected on the results of RNA works.

In conclusion, the stroma-rich chordoma is well-enhanced on MRI and has transcriptomically more predominant EMT features that implies more aggressive biologic behavior. Further study with a larger cohort and sufficient tumor sample would verify the clinical significance of stromal component in chordoma.

## Materials and methods

### Study population

Electronic medical records were reviewed for patients with pathologically confirmed skull base chordomas treated at our institution between May 2011 and March 2020. Patients were included in the study only if the following were available for review: pretreatment MRI examination, operative reports, pathologic reports and available tumor specimens. Using these inclusion and exclusion criteria, a total of 45 patients were included in this study.

### Clinical data evaluation

Clinical data for the 45 patients were obtained including documentation of the extent of surgical resection, type of adjuvant treatment, progression or recurrence of tumor, follow up period. The extent of surgical resection was categorized as gross total or subtotal based on surgical and radiologic assessments documented in operative reports and immediate postoperative imaging. Progression and recurrence were evaluated by imaging assessments, with all cases except one whose progression/recurrence was pathologically confirmed by repeat resection.

### Pathologic evaluation

**Figure 6:**
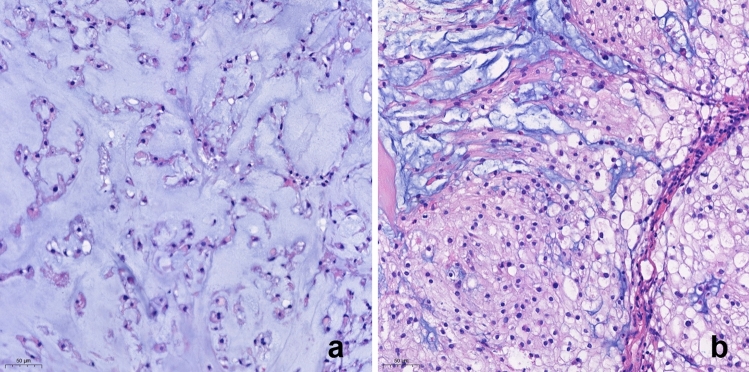
Histology of stroma-rich (**a**) and stroma-poor (**b**) chordomas. (**a**) Stroma-rich chordoma shows extensive myxoid stroma with a few strands of tumor cells. (**b**) Stroma-poor chordoma shows a diffuse nodular proliferation of tumor cells with scant myxoid stroma.

All available hematoxylin and eosin-stained slides were reviewed by a neuropathologist (YJC) who was blind to the radiologic features or prognosis. BNCT-like feature was defined as sheets of large uni- or multivacuolated adipocyte-like cells with eccentrically located small nuclei and lacked the intervening tumor matrix. Myxoid stroma as well as tumor cells was considered as the tumor area. Tumor cellularity was defined as the percentage of the area occupied by tumor cells within the whole tumor area. Chordomas with > 60% of stroma (< 40% cellularity) in the tumor area were defined as stroma-rich (Fig. 6a), and the remaining were classified as stroma-poor chordoma (Fig. 6b).

### MRI evaluation

The MRI scans were evaluated by an experienced neuroradiologist who was blind to the reviewed histologic diagnosis or prognosis. MRI examinations were performed on a 3T MRI scanner. The available MRI sequences were variable, but axial T2-weighted and pre- and post-contrast T1-weighted images were available in all cases. Homogeneity of T2-weighted signal intensity was evaluated as either homogeneous or heterogeneous compared with the signal intensity of intact bone marrow. T2-weighted MR SI on each lesion was made based on high, intermediate, or low signal intensity compared with the signal intensity of intact bone marrow. Contrast-enhanced T1-weighted signal intensity on each lesion was defined as the presence or absence of enhancement compared with unenhanced T1-weighted images^34^.

### RNA sequencing and data analysis

Of the 45 chordoma cases, 10 were selected for RNA sequencing: Four (40%) had rich stroma, whereas six (60%) had sparse stroma.

RNA was quantified using Qubit®2.0 (Life Technologies, Burlington, Canada) and qualified using an Agilent RNA 6000 Nano Kit and the 2100 Bioanalyzer (Agilent Technologies, Palo Alto, CA, USA). RNA libraries were prepared using TruSeq Stranded Total RNA Kit (20020596, Illumina, San Diego, CA, USA) according to the manufacturer’s instructions. Briefly, rRNA was depleted from 100 ng of total RNA, fragmented, and primed for cDNA synthesis. After synthesis of the first and second strand cDNA, libraries were constructed for Illumina Paired-End Sequencing. Fragment sizes for all libraries were measured using the 2100 Bioanalyzer (Agilent Technologies), and quantitative polymerase chain reaction was performed on the LightCycler® 480 System (Roche, CA, USA) with the KAPA library quantification kit (KK4854; Kapa Biosystems, Wilmington, MA, USA). Sequencing was performed on an Illumina Hiseq2500 and produced on average a total of 100 million 100 bp reads per sample. Raw sequencing data were processed with Trimmomatic (version 0.39) to remove remaining bases from adaptors and low-quality bases^35^. The expression of transcripts were quantified using the Salmon software package^36^ (version 1.7.0) with the Gencode v39(GRCh38.p13)^37^. Then, the transcript-level expression data were summarized to the gene-level expression by using tximport^38^. Low expressed genes whose average reads count across samples are less than 10 were removed. Identification of differentially expressed genes between the stomal-poor and stroma-rich group were performed for the remained 23,493 genes using DEseq2 package^39^. PCA using the most variable 500 genes across the samples was also performed to check whether there is any outlier sample in the gene expression dataset.

### Quantification of mRNA expression

After RNA sequencing for 10 samples, mRNA expression of differentially expressed genes was further measured using available 30 samples in remainders (18 stroma-rich and 12 stroma-poor tumors. Taqman Gene Expression Assays (Thermo Fisher Scientific, Waltham, MA, USA) were performed to quantify the expression of *NDRG4* (Hs00224735_m1)*, SLC25A16* (Hs01039823_m1), *CAMK2D* (Hs00943538_m1), *UBE2D1* (Hs00696921_m1), *ACTB* (Hs99999903_m1), *SFRP2* (Hs01564480_m1), *SERPINE2* (Hs00299953_m1, *COCH* (Hs00990772_m1), *ITGA2B* (Hs01116228_m1) and *GAPDH* (Hs04260367_gH). cDNA was produced using the Superscript™|| RT-PCR System (Invitrogen, Karlsruhe, Germany) according to the manufacture’s recommendations for oligo(dT)20 primed cDNA-synthesis. cDNA synthesis was performed on 500 ng of RNA, at 42 °C. Quantitative TaqMan PCR was performed in a ABI PRISM 7900HT Sequence Detection System (Applied Biosystems, Foster City, CA, USA) in 384-well microtiter plates using a final volume of 10 μl. Optimum reaction conditions were obtained with 5 μl of Universal Master Mix (Applied Biosystems) containing dNUTPs, MgCl_2_, reaction buffer and Ampli Taq Gold, 90 nM of primer(s) and 250 nM fluorescence-labeled TaqMan probe. Finally, 2 μl template cDNA was added to the reaction mixture. The primer/TaqMan probe combinations were designed on each target sequences. Amplifications were performed starting with a 10minunte-template denaturation step at 95 °C, followed by 40 cycles at 95 °C for 15 s and 60 °C for 1 min. All samples were amplified on triplicate and data were analyzed with Sequence Detector software (Applied Biosystems). The delta C_T_ valule is determined by subtracting the average endogenous control C_T_ value from the individual C_T_ value of target gene.

### Statistical analysis

‘DESeq2’ was used to calculate the differential gene expression from the gene level read count table obtained from the analysis of RNA sequencing data using Salmon and tximport. The rows and columns of the gene level read count table corresponds to the genes and samples, respectively. The log2 fold change (log_2_FC) and *p*-value of differential gene expression between the two groups was calculated using the apeglm method from the ‘DESeq2’ package^40^. Differentially expressed genes were visualized with a volcano and heatmap plot using the ‘EnhancedVolcano’ and a ‘pheatmap’ packages, respectively. Gene set analysis of differentially expressed genes on gene ontology terms was performed with the gseGO function from the ‘clusterProfiler’ R Package^17,41^. The gseGO function assess gene ontology (GO) terms whether there is significant overlap between the genes of each GO term related and a list of genes differentially expressed. Clinical data and radiologic findings were analyzed using SPSS (version 25.0). Statistical significance was set at *p*-value < 0.05.

### Ethics declarations

All procedure was performed in accordance with the 1964 Declaration of Helsinki and its later amendments or comparable ethical standards. This retrospective study was approved by the Institutional Review Board of Gangnam Severance Hospital, Seoul, South Korea (No. 3-2020-0338). The need for informed consent was waived by the review board.

## Supplementary Information

Supplementary Tables.

Supplementary Figure 1.

Supplementary Figure 2.

Supplementary Figure 3.

## Data Availability

The datasets generated during and/or analyzed during the current study are available from the corresponding author on reasonable request.

## References

[1] Vujovic S (2006). Brachyury, a crucial regulator of notochordal development, is a novel biomarker for chordomas. J. Pathol..

[2] Meis JM, Raymond AK, Evans HL, Charles RE, Giraldo AA (1987). "Dedifferentiated" chordoma. A clinicopathologic and immunohistochemical study of three cases. Am. J. Surg. Pathol..

[3] Bisceglia M, D'Angelo VA, Guglielmi G, Dor DB, Pasquinelli G (2007). Dedifferentiated chordoma of the thoracic spine with rhabdomyosarcomatous differentiation. Report of a case and review of the literature. Ann. Diagn. Pathol..

[4] Mobley BC (2010). Loss of SMARCB1/INI1 expression in poorly differentiated chordomas. Acta Neuropathol..

[5] Tarpey PS (2017). The driver landscape of sporadic chordoma. Nat. Commun..

[6] Antonelli M (2017). SMARCB1/INI1 involvement in pediatric chordoma: A mutational and immunohistochemical analysis. Am. J. Surg. Pathol..

[7] Shih AR (2018). Clinicopathologic characteristics of poorly differentiated chordoma. Mod. Pathol..

[8] Hoch BL, Nielsen GP, Liebsch NJ, Rosenberg AE (2006). Base of skull chordomas in children and adolescents: A clinicopathologic study of 73 cases. Am. J. Surg. Pathol..

[9] Hasselblatt M (2016). Poorly differentiated chordoma with SMARCB1/INI1 loss: A distinct molecular entity with dismal prognosis. Acta Neuropathol..

[10] McMaster ML, Goldstein AM, Bromley CM, Ishibe N, Parry DM (2001). Chordoma: Incidence and survival patterns in the United States, 1973–1995. Cancer Causes Control.

[11] Chugh R (2007). Chordoma: The nonsarcoma primary bone tumor. Oncologist.

[12] Tzortzidis F, Elahi F, Wright D, Natarajan SK, Sekhar LN (2006). Patient outcome at long-term follow-up after aggressive microsurgical resection of cranial base chordomas. Neurosurgery.

[13] Colli B, Al-Mefty O (2001). Chordomas of the craniocervical junction: Follow-up review and prognostic factors. J. Neurosurg..

[14] Wu Z (2010). Prognostic factors for long-term outcome of patients with surgical resection of skull base chordomas-106 cases review in one institution. Neurosurg. Rev..

[15] Bohman LE, Koch M, Bailey RL, Alonso-Basanta M, Lee JY (2014). Skull base chordoma and chondrosarcoma: Influence of clinical and demographic factors on prognosis: A SEER analysis. World Neurosurg..

[16] Zou MX (2018). Prognostic factors in skull base chordoma: A systematic literature review and meta-analysis. World Neurosurg..

[17] Sze G (1988). Chordomas: MR imaging. Radiology.

[18] Tian K (2017). MR imaging grading system for skull base chordoma. Am. J. Neuroradiol..

[19] Lin E, Scognamiglio T, Zhao Y, Schwartz TH, Phillips CD (2018). Prognostic implications of gadolinium enhancement of skull base chordomas. Am. J. Neuroradiol..

[20] Zou MX (2019). The relationship between tumor-stroma ratio, the immune microenvironment, and survival in patients with spinal chordoma. Neurosurgery.

[21] Tirabosco R (2008). Brachyury expression in extra-axial skeletal and soft tissue chordomas: A marker that distinguishes chordoma from mixed tumor/myoepithelioma/parachordoma in soft tissue. Am. J. Surg. Pathol..

[22] Presneau N (2011). Role of the transcription factor T (brachyury) in the pathogenesis of sporadic chordoma: A genetic and functional-based study. J. Pathol..

[23] Yang XR (2009). T (brachyury) gene duplication confers major susceptibility to familial chordoma. Nat. Genet..

[24] Rosenberg AE, Brown GA, Bhan AK, Lee JM (1994). Chondroid chordoma—A variant of chordoma. A morphologic and immunohistochemical study. Am. J. Clin. Pathol..

[25] La Corte E (2021). Peri-operative prognostic factors for primary skull base chordomas: Results from a single-center cohort. Acta Neurochir. (Wien).

[26] Li Q (2018). HSCs-derived COMP drives hepatocellular carcinoma progression by activating MEK/ERK and PI3K/AKT signaling pathways. J. Exp. Clin. Cancer Res..

[27] Vincourt JB (2008). Increased expression of matrilin-3 not only in osteoarthritic articular cartilage but also in cartilage-forming tumors, and down-regulation of SOX9 via epidermal growth factor domain 1-dependent signaling. Arthritis Rheum..

[28] Buchholz M (2003). SERPINE2 (protease nexin I) promotes extracellular matrix production and local invasion of pancreatic tumors in vivo. Cancer Res..

[29] Navab R (2016). Integrin alpha11beta1 regulates cancer stromal stiffness and promotes tumorigenicity and metastasis in non-small cell lung cancer. Oncogene.

[30] Wang Y (2017). Amino acid transporter SLC38A3 promotes metastasis of non-small cell lung cancer cells by activating PDK1. Cancer Lett..

[31] Shi L, Resaul J, Owen S, Ye L, Jiang WG (2016). Clinical and therapeutic implications of follistatin in solid tumours. Cancer Genomics Proteomics.

[32] Fantozzi A (2014). VEGF-mediated angiogenesis links EMT-induced cancer stemness to tumor initiation. Can. Res..

[33] Masiulionyte B, Valiulyte I, Tamasauskas A, Skiriute D (2019). Metallothionein genes are highly expressed in malignant astrocytomas and associated with patient survival. Sci. Rep..

[34] Nishiguchi T (2011). Differentiating benign notochordal cell tumors from chordomas: Radiographic features on MRI, CT, and tomography. Am. J. Roentgenol..

[35] Bolger AM, Lohse M, Usadel B (2014). Trimmomatic: A flexible trimmer for Illumina sequence data. Bioinformatics.

[36] Patro R, Duggal G, Love MI, Irizarry RA, Kingsford C (2017). Salmon provides fast and bias-aware quantification of transcript expression. Nat. Methods.

[37] Frankish A (2019). GENCODE reference annotation for the human and mouse genomes. Nucleic Acids Res..

[38] Soneson C, Love MI, Robinson MD (2015). Differential analyses for RNA-seq: Transcript-level estimates improve gene-level inferences. F1000Research.

[39] Love MI, Huber W, Anders S (2014). Moderated estimation of fold change and dispersion for RNA-seq data with DESeq2. Genome Biol..

[40] Zhu A, Ibrahim JG, Love MI (2019). Heavy-tailed prior distributions for sequence count data: Removing the noise and preserving large differences. Bioinformatics.

[41] Yu G, Wang LG, Han Y, He QY (2012). clusterProfiler: An R package for comparing biological themes among gene clusters. OMICS.

