# Supplementary material for: Differences in stromal component of chordoma are associated with contrast enhancement in MRI and differential gene expression in RNA sequencing
Source: Sci Rep. 2022 Oct 3;12:16504. doi: 10.1038/s41598-022-20787-3 (PMC9529962; doi:10.1038/s41598-022-20787-3)
Supplement: Supplementary file 1 — Supplementary Tables. [file 41598_2022_20787_MOESM1_ESM.docx]

**Supplementary Table 1.** Patient characteristics according to the stromal component of chordomas.

| **Variables** | **Whole (n = 45)** | **Stroma-poor (n = 20)** | **Stroma-rich (n = 25)** | ***p*-value** |
| --- | --- | --- | --- | --- |
| Age, year (mean) | 47.4±14.4 | 48±14.9 | 46.7±14.2 | 0.727 |
| Sex, female | 25 (55.6%) | 10 (50.0%) | 15 (60.0%) | 0.502 |
| Tumor size, mm (mean ± SD) | 32.3±19.0 | 34.5 ± 22.5 | 35.9±16.3 | 0.810 |
| Progression (n = 43) | 15 (37.5%) | 5 (25.0%) | 10 (40.0%) | 0.251 |
| F/U period, days (median) | 621 (IQR 270.5–1768.3) | 543.5 (187.3–1953.3) | 632.5 (302.0–1653.3) | 0.778 |
| Resection type (n = 44) |  |  |  |  |
| Subtotal removal | 16 | 7 | 9 |  |
| Total removal | 28 | 13 | 15 |  |
| Adjuvant treatment* |  |  |  |  |
| None | 3 | 2 | 1 |  |
| Photon therapy | 30 | 13 | 17 |  |
| Proton therapy | 17 | 7 | 10 |  |

SD, standard deviation; F/U, follow-up; IQR, interquartile range

**Supplementary Table 2.** Gene ontology terms overrepresented in the set of differentially expressed genes.

| **Overlapped genes with DEGs** | **GO ID** | **adjusted *p*-value** | **q-value** |
| --- | --- | --- | --- |
| SFRP2/COMP/ITGA11/MATN3/ADAMTS16/COL11A2/COL9A2/COL16A1/COL9A3/BGN/ADAMTS6/LOXL2/ITGA2B | GO:0030198: extracellular matrix organization  GO:0043062: extracellular structure organization  GO:0045229: external encapsulating structure organization | <0.001 | <0.001 |
| SFRP2/SERPINE2/COMP/MATN3/COCH/COL11A2/COL9A2/COL16A1/COL9A3/BGN/LOXL2 | GO:0062023: collagen-containing extracellular matrix | <0.001 | <0.001 |
| SFRP2/COMP/MATN3/CNMD/COL11A2/BGN/LOXL2 | GO:0051216: cartilage development  GO:0061448: connective tissue development | <0.001 | <0.001 |
| COMP/MATN3/COL11A2/COL9A2/COL16A1/COL9A3/BGN | GO:0005201: extracellular matrix structural constituent | <0.001 | <0.001 |
| COL11A2/COL9A2/COL16A1/COL9A3/C1QL1 | GO:0005581: collagen trimer | <0.001 | <0.001 |
| SERPINE2/COMP/FST/ACADL/FGFR1 | GO:1901681: sulfur compound binding | 0.012 | 0.009 |
| COL11A2/COL9A2/COL16A1/COL9A3 | GO:0030020: extracellular matrix structural constituent conferring tensile strength | <0.001 | <0.001 |
| SFRP2/COMP/COL11A2/LOXL2 | GO:0030199: collagen fibril organization | 0.003 | 0.003 |

**Supplementary Figure legends**

**Supplementary Figure 1.** Survival curve of chordoma patients based on the stromal proportion

Progression-free survival of stroma-rich chordoma appears inferior compared to that of stroma-poor chordoma, but statistically insignificant.

**Supplementary Figure 2.** Principal component analysis plot using the 500 most variable genes

Principal component analysis plot using the 500 most variable genes reveals no significant bias from the dataset.

**Supplementary Figure 3.** Quantitative mRNA expression analysis.

Based on the result of RNA sequencing, mRNA expression analysis for further validation shows tendency of increased (a and b) and decreased (c and d) of mRNA expression in stroma-rich chordoma compared to that of stroma-poor chordoma.
