# Supplementary figures and images for: Differences in stromal component of chordoma are associated with contrast enhancement in MRI and differential gene expression in RNA sequencing
Source: Sci Rep. 2022 Oct 3;12:16504. doi: 10.1038/s41598-022-20787-3 (PMC9529962; doi:10.1038/s41598-022-20787-3)

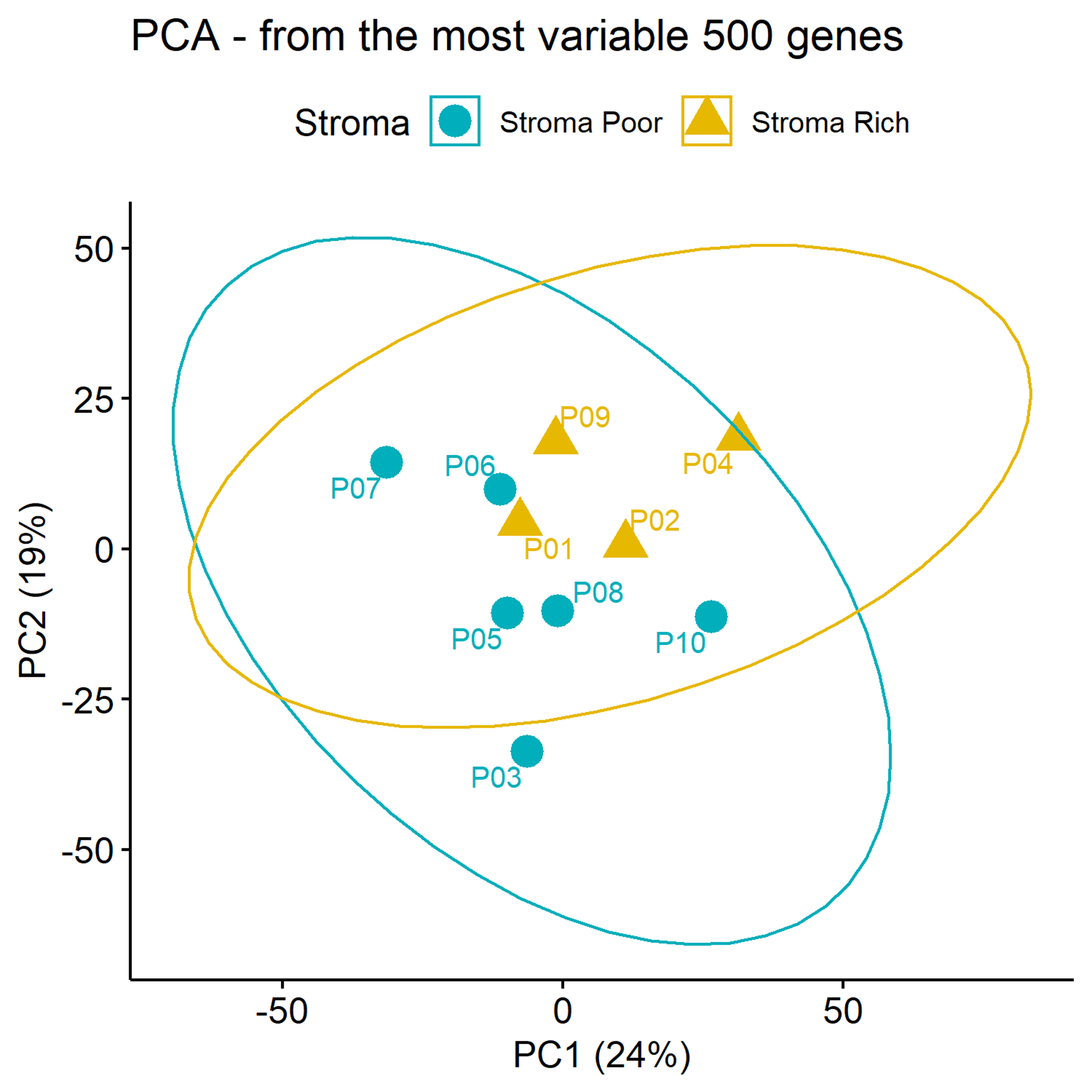

Supplement: Supplementary file 3 — Supplementary Figure 2. [file 41598_2022_20787_MOESM3_ESM.tif]

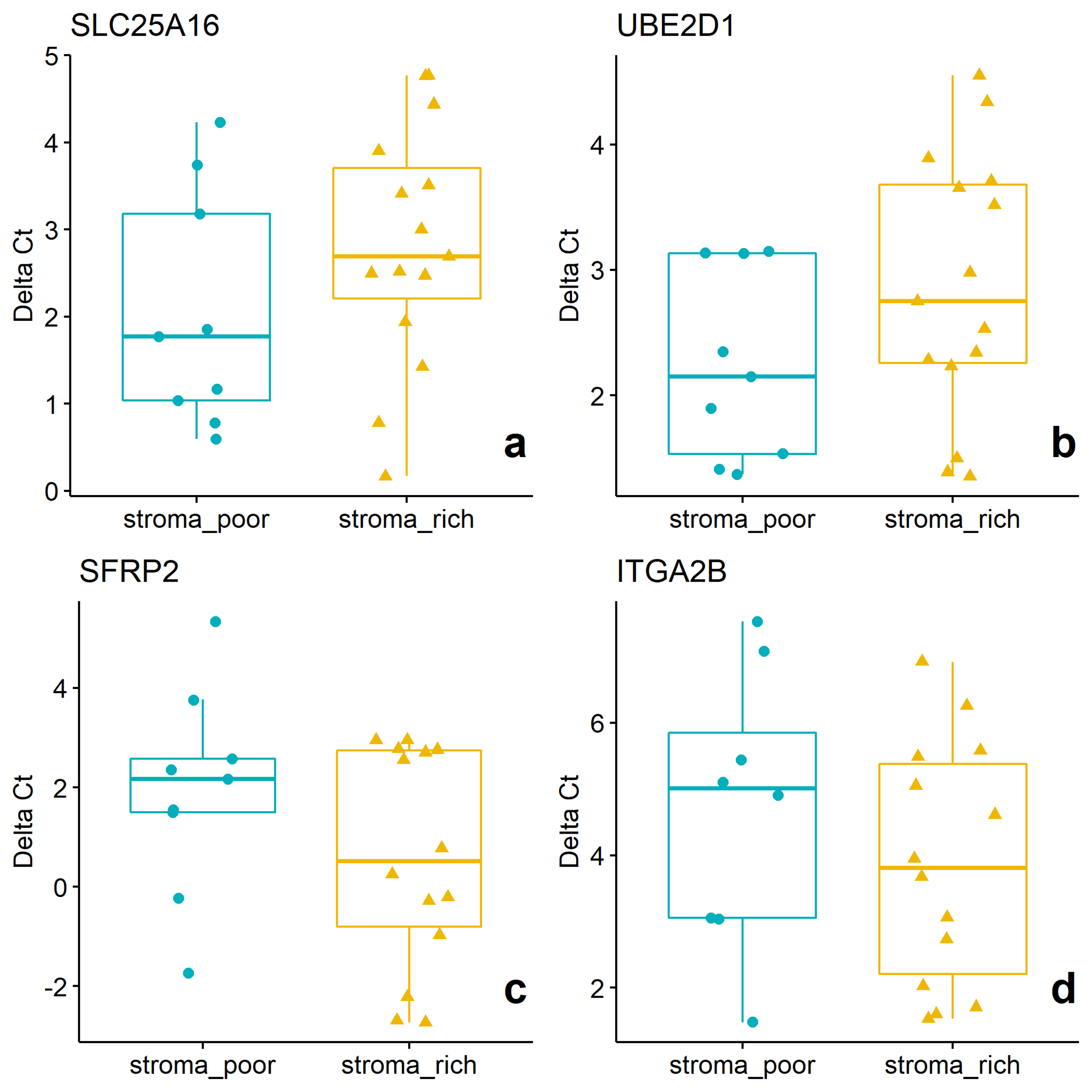

Supplement: Supplementary file 4 — Supplementary Figure 3. [file 41598_2022_20787_MOESM4_ESM.tif]
